# An innovative, digital approach to training district health care providers on essential newborn care skills: findings from a pilot cluster-randomised trial in Lao People’s Democratic Republic

**DOI:** 10.7189/jogh.15.04163

**Published:** 2025-06-02

**Authors:** Sayaka Horiuchi, Rachel Smith, Bouasengnignom Phrasithideth, Outhevanh Kounnavongsa, Chitsana Thilakoune, Hongkham Xayavong, Kentaro Sakamaki, Sommana Rattana, Joshua P Vogel

**Affiliations:** 1 Burnet Institute, Women’s, Children’s and Adolescents’ Health Program, Melbourne, Australia; 2 Japan Society for the Promotion of Science, Overseas Research Fellowship, Tokyo, Japan; 3 University of Yamanashi, Department of Epidemiology and Environmental Medicine, Yamanashi, Japan; 4 Mahosot Hospital, Vientiane, Lao PDR; 5 World Health Organization, Vientiane, Lao PDR; 6 Retired midwife, Mahosot hospital, Vientiane, Lao PDR; 7 Lao Tropical and Public Health Institute, Vientiane, Lao PDR; 8 Juntendo University, Faculty of Health Data Science, Tokyo, Japan; 9 Ministry of Health, Department of Health Care and Rehabilitation, Vientiane, Lao PDR

## Abstract

**Background:**

In Lao People’s Democratic Republic, the skills of newborn care providers at district hospitals degrades soon after training due to lack of regular supervision and/or refresher training. This pilot trial evaluated the potential effectiveness and acceptability of the novel intervention to maintain providers’ early essential newborn care (EENC) knowledge and skills.

**Methods:**

This was a parallel, cluster-randomised pilot trial with embedded focus group discussions in Lao People’s Democratic Republic. Newborn care providers at four district hospitals (clusters) in two provinces participated. The four clusters were randomised into two arms, within each province. Both arms initially received standard EENC coaching. The intervention arm used repeated self-practise combined with mobile phone-based supportive supervision from EENC facilitators, while the control arm received no additional support. The primary outcome, a standardised assessment of provider’s EENC knowledge and skills measured at three months post-randomisation, was analysed by intention-to-treat. Qualitative data underwent thematic analysis.

**Results:**

Forty-four providers were recruited: 22 in each arm. Thirty-seven providers and 19 facilitators completed data collection and participated in focus group discussions. The knowledge and skill scores at three months were 0.59 points (95% confidence interval (CI) = −1.40, 2.52) and 3.67 points (95% CI = −2.90, 10.24) higher in the intervention arm than in the control arm, after adjusting for baseline scores. Both providers and facilitators viewed the intervention positively, though time constraints limited self-practise sessions. While they believed it could improve their clinical performance, confidence in sustaining the intervention was low.

**Conclusions:**

Though not powered for statistical significance, this study showed the intervention may help maintain newborn care skills and was well accepted. A definitive trial is warranted to evaluate its effectiveness on clinical practice and health outcomes, along with further investigation into factors that support sustainability.

**Registration:**

This trial was registered with the Australian and New Zealand Clinical Trial Registry (ACTRN12623000957695).

Poor-quality care provision is a key barrier to reducing newborn mortality [1]. In low- and middle-income countries (LMICs), poor quality care is the cause of more newborn deaths than non-utilisation of the health system [2]. Ensuring newborn care providers have adequate knowledge and skills on essential newborn care is a critical step to prevent newborn morbidity and mortality. However, the knowledge and skills of providers deteriorates over time, especially without regular practice [3]. To ensure that good-quality newborn care is universally available, providers need continual in-service training and practice opportunities.

Early Essential Newborn Care (EENC) is a package of interventions that prevent newborn deaths. It was developed by the World Health Organization (WHO) Regional Office for the Western Pacific and is widely used in the region [4]. In countries implementing EENC, reductions in Neonatal Intensive Care Unit admissions and increased breastfeeding rates were observed [5,6]. However, a 2017 study in Lao People’s Democratic Republic (Lao PDR) found that provider’s EENC skills degraded quickly after an initial EENC coaching session [7]. This skill degradation was most evident in the management of non-breathing babies. This is a particular problem in facilities with low birth volumes, where management of a non-breathing baby may occur rarely [8].

These shortfalls in newborn care provision require new approaches to staff training. For example, a ‘low-dose, high-frequency’ approach – using short, targeted, repeated, in-service, simulation-based learning activities – has proven effective in helping staff retain clinical knowledge and skills in several different professional disciplines and in LMIC settings [9,10]. Combining training with repeated follow-up supervisory visits improves provider skills, through providing motivation to improve [11,12]. Currently, in Lao PDR, newborn care providers at a district hospital cannot expect to receive a supervisory visit more than once a year due to limited human resources and travel budgets [3]. Digital technologies can help fill these gaps, by extending access to clinical training for health care providers [13]. These technologies are particularly helpful in remote or hard-to-reach areas with internet access.

Our scoping review on digital technologies to improve newborn care training found that eLearning platform and mobile applications were often used in LMICs and that some of these approaches were effective in improving clinical skills [14]. On the other hand, the effectiveness of the self-learning approach depends on the intensity of learner participation. Regular external visits are a key to sustaining learner participation [7]. To integrate a self-learning and a frequent external supervision, we developed a novel intervention in Lao PDR. In this intervention, routine EENC refresher coaching is followed by repeated, self-directed, practise sessions by district health care providers, with mobile phone-based supportive supervision from EENC facilitators. The intervention was co-developed with key stakeholders, including government officials at Ministry of Health, midwives, nurses and paediatricians. This pilot trial aimed to evaluate potential effectiveness, feasibility and acceptability of this intervention in maintaining providers’ EENC knowledge and skills, to inform a future definitive trial.

## METHODS

The protocol was prospectively registered with the Australia and New Zealand Clinical Trial Registry (ACTRN12623000957695) [15]. We report the findings according to the Consolidated Standards of Reporting Trials (CONSORT) extension to randomised pilot and feasibility trials [16].

### Study setting

Lao PDR consists of one capital city (Vientiane Capital) and 17 provinces; each province is divided into several districts. Six central hospitals are in Vientiane Capital. Each province has a provincial hospital, which oversees all district hospitals within that province. District hospitals provide primary to secondary-level care, either with surgical facilities (type A district hospital) or without (type B). District hospitals do not have a neonatal intensive care unit; newborns requiring intensive care are transferred to provincial or central hospitals. Newborn care providers (midwives, nurses, obstetricians or paediatricians) from central and provincial hospitals have been trained as EENC facilitators; they oversee and support the EENC rollout in district hospitals. This study was conducted in Huaphanh and Khammouane provinces of Lao PDR. These two provinces were selected as provincial EENC facilitators have been actively engaged in rollout activities and are well-equipped to supervise and assess EENC practices in district hospitals. Neonatal mortality rates for these two provinces were higher than the national average in the 2017 survey [17]. Although this study only focused on two provinces, the environment may be similar throughout the country. At the district level, we can expect good mobile network coverage and district hospitals with little variation in facilities and health system structure.

### Study design

This was a cluster-randomised pilot trial with an embedded qualitative study. We used a two-arm, parallel-group design, with one stratum based on province. Clusters (district hospitals) were allocated in a 1:1 ratio within the same stratum (province). A cluster design was used as it would not be practical to randomise individual providers within a single hospital due to the nature of the intervention (group practise) and the risk of cross-contamination. Group interaction can help increase motivation and prolong engagement [18], hence we encouraged providers at the same facility to continually implement self-practise together. To explore acceptability, enablers, and barriers to the intervention, we conducted a qualitative descriptive study using focus group discussions (FGDs). This was based on the paradigm of constructivism, to understand socially constructed perspectives and realities around the implementation of the intervention [19].

### Participants

Participating clusters were district hospitals in Huaphanh and Khammouane provinces. We selected two district hospitals from each province that were similar in terms of distance from the provincial capital and number of annual births. We obtained formal permission from the leadership of these four district hospitals, the two associated provincial hospitals, and the Ministry of Health. Eligible participants at the four district hospitals were newborn care providers – this includes doctors, nurses, midwives and medical assistants. Trained research staff obtained written informed consent before commencement.

### Randomisation and masking

Clusters were stratified by province. A random number sequence was generated within each stratum by a trial statistician. A different researcher enrolled district hospitals and randomly assigned one district hospital in each stratum to the intervention arm and the other to the control arm. Allocation was performed prior to the commencement of the EENC coaching visit by EENC facilitators. STATA/MP 17.0 for Windows (StataCorp LLC, Texas, USA) was used to generate the random sequence. Due to the nature of the intervention, masking of participants and data collectors was not possible.

### Intervention and control

Intervention and control clusters received a standard, one-day refresher EENC coaching by trained, certified EENC facilitators. This includes lectures and simulated practise of breathing and non-breathing babies. The 21 EENC facilitators were from central (seven) and provincial (14) hospitals; those from central hospitals oversaw the provincial-level facilitators. Baseline knowledge and skill assessments were completed by all providers immediately after the EENC coaching.

**Figure 1:**
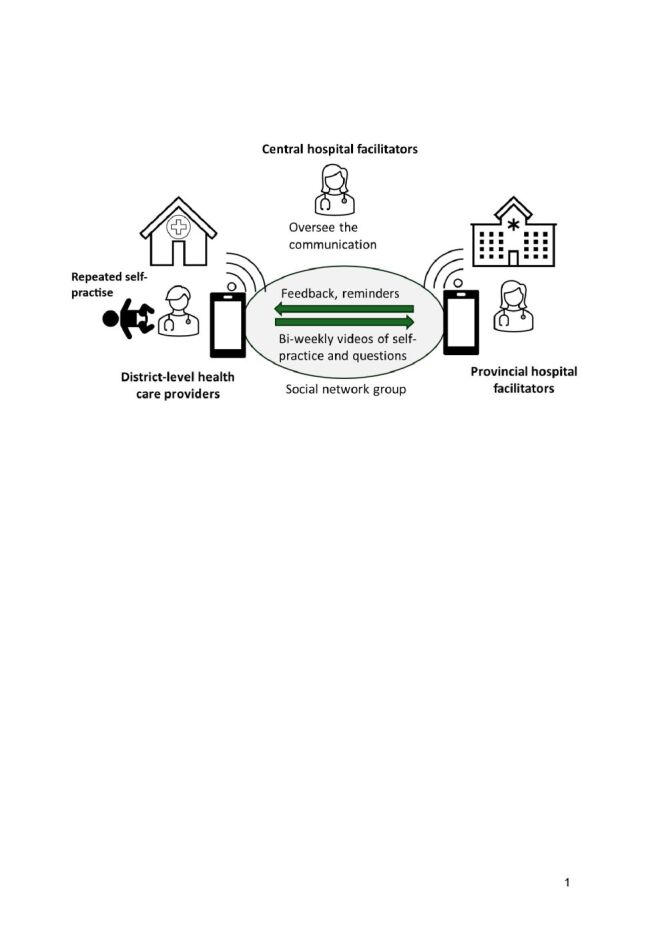
Intervention.

After the EENC coaching, providers in the intervention arm were taught how to implement self-practise and receive mobile phone-based supportive supervision. They practised taking and sending a video via a group social networking service (WhatsApp), which is widely used for communication in Lao PDR. A WhatsApp group was established for each district hospital in the intervention arm for sharing videos. The group included EENC facilitators and all participating providers at that district hospital. Providers were expected to conduct self-practise of their EENC skills (management of both breathing and non-breathing baby) once every two weeks using simulation equipment alongside their clinical duties (**Figure 1**). They were encouraged to send videos of these practise sessions to EENC facilitators fortnightly, via the WhatsApp group. The facilitators were expected to review the videos and share feedback via this group. Participating providers in control hospitals were advised to continue their usual practice, but no further support was provided after the one-day EENC coaching.

### Study outcomes and data collection

We collected demographic data on providers using a questionnaire at baseline. The primary outcomes were provider’s knowledge and skills on EENC. Within the skills outcome, we also assessed separately skills for managing a breathing baby and a non-breathing baby. Data were collected twice in both arms – immediately after the refresher EENC coaching (baseline), and three months after the EENC coaching (endline). Knowledge and skills on EENC were measured via a paper test and simulation test, respectively – these are standardised assessments developed by WHO and the Lao PDR Ministry of Health [20]. The simulation test assessed essential neonatal care skills, including achieving a chest rise within one minute of birth for a non-breathing baby. If a particular skill was completed, it was given a score of two and if it was partially completed, a score of one. The maximum total scores for knowledge (total), skills (total), skill (breathing baby) and skill (non-breathing baby) are 18, 106, 44 and 62, respectively. Implementation status of the intervention was measured by counting videos shared in the WhatsApp groups, and a weekly online survey for providers to self-report their practise. The survey collected self-reported data on time spent practising.

We conducted six FGDs at endline: one with central-level facilitators, two with provincial-level facilitators (one per province), and three with providers from participating district hospitals. All FGDs were conducted in Lao language, and were facilitated by a trained, independent researcher. These FGDs used a semi-structured guide based on the Theoretical Framework of Acceptability [21]. Each FGD lasted approximately 60 minutes, were audio-recorded, transcribed in Lao and translated into English.

### Sample size and power calculation

This pilot trial aimed to explore feasibility of a novel intervention, and thus was not powered to detect a definitive difference in the primary outcomes. In planning this trial, we aimed to recruit all providers in the four clusters, which varied from 12–14 per cluster (53 in total). Assuming a cluster size of 13 and an inter-cluster correlation of 0.08, we estimated a detectable effect size of 1.7 (9.5 points difference in a total skill score) with a two-sided type I error rate of 5 and 80% power. We used a standard deviation (SD) of 5.6 for the simulation test, based on findings of a 2017 cluster trial in Lao PDR [7].

### Data analysis

Characteristics of participating providers were summarised using means and SDs, or numbers and percentages, stratified by trial arm. We tested for differences in the baseline characteristics of participants between the two arms using the Wald test. The primary outcomes – provider knowledge and skills scores – were summarised as means and SDs by arm. Additionally, we calculated the percentage of providers who could achieve a chest rise within one minute of birth in the management of a non-breathing baby.

The primary outcomes were analysed by intention-to-treat. We estimated the intervention’s effects using a linear mixed effects model employing the maximum likelihood method. The model was adjusted for baseline scores. We treated the intervention as a fixed effect, and variation within a district hospital as a random effect. As the mixed model analysis removes participants with only a baseline value [22], to follow the intention-to-treat principle, we also performed analysis using: 1) alternative mixed methods without imputation

2) single imputation, using the baseline value carried forward

3) multiple imputation using predictive mean matching.

In the alternative mixed methods, interaction between the intervention and time (baseline and endline) was estimated. In the multiple imputation, we conducted 20 imputations by including the corresponding baseline value of the primary outcome and the intervention value. A sensitivity analysis was performed by adjusting for the baseline variables that showed significant differences between the two arms and could conceivably be a prognostic factor for a primary outcome [23]. A significance level of 5% with two tailed was applied for all significance analyses. All statistical analyses were performed using STATA/MP 17.0 for Windows (StataCorp LLC, Texas, USA).

### Qualitative analysis

We analysed FGD data using thematic analysis according to Braun & Clarke’s six-phase framework [24]. One researcher (SH) reviewed each transcript and generated codes. The codes were inductively analysed and mapped to the constructs of the Theoretical Framework of Acceptability [21], facilitators and barriers to the implementation. Triangulation was conducted through discussing the results with other two researchers (RS, JPV) who had expertise in provider training and perinatal care in resource-limited settings. Member checks were carried out with representatives of the participants to ensure that the content and interpretation of the data were correct. NVivo 14.23.3 (61) (Lumivero, Denver, CO, USA) was used to organise the codes.

## RESULTS

### Study participants

**Figure 2:**
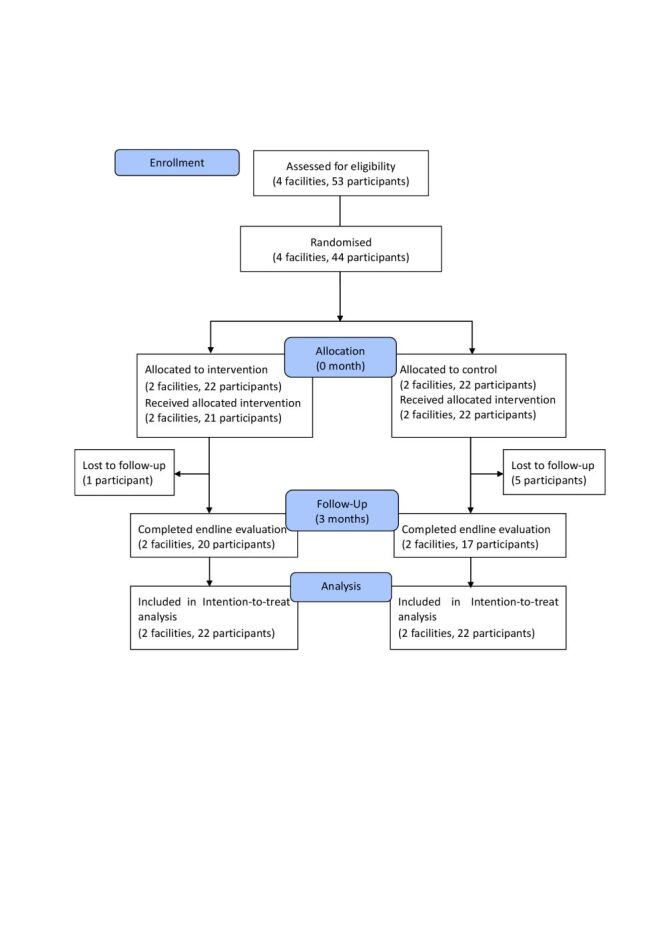
Flow diagram.

The study was conducted between October 2023–February 2024. Four district hospitals were randomised, two in each arm. Among 53 potential participants, nine health care providers were not available on the day the facilitators and research team visited. We recruited 22 eligible health care providers in both arms (**Figure 2**). One participant in the intervention arm did not complete the allocated intervention. Six providers were unable to attend the final evaluation and were lost to follow up (one in intervention, five in control). In total, 20 providers in the intervention arm and 17 in the control arm completed endline data collection. Twenty district providers from the intervention arm, 14 provincial facilitators and five central facilitators participated in FGDs.

The baseline characteristics of providers for the two arms were comparable for age, gender, cadre, ethnicity, and level of confidence in supporting childbirth (**Table 1**). The number of births attended per month was higher in intervention (mean (x̄) = 7.8, SD = 4.4) than control (x̄ = 4.4, SD = 3.0, *P* < 0.005). Mean years of maternity care experience was lower in intervention (x̄ = 9.0, SD = 5.5) than control (x̄ = 13.3, SD = 10.4, *P* = 0.081). The mean knowledge and total skill scores at baseline were x̄ = 14.8, SD = 2.3; and x̄ = 100, SD = 4.5 in the intervention arm and x̄ = 16.4, SD = 1.4 and x̄ = 100.6, SD = 4.2 in the control arm (**Table 2**). There were no statistical differences in baseline scores between the arms (*P* = 0.153 and *P* = 0.770, respectively). For the management of a non-breathing baby, the proportion of chest rise achievement within one minute of birth at baseline was 57.1% in the intervention arm, and 54.6% in the control arm. The proportion at endline was 10.0% in the intervention arm and 0.0% in the control arm.

**Table 1:** Baseline characteristics (n = 44)

Variables	Intervention (n = 22)	Control (n = 22)	*P*-value (Wald test)
Age (in years), x̄ (SD)	34.8 (9.1)	36.2 (9.2)	0.855
*<35*	13 (59.1)	9 (40.9)	0.642
*≥35*	9 (40.9)	13 (59.1)	
Gender, n (%)			
*Female*	17 (77.3)	17 (77.3)	1.000
*Male*	5 (22.7)	5 (22.7)	
Ethnicity, n (%)			
*Lao Loun*	15 (68.2)	20 (90.9)	0.339
*Others*	7 (31.8)	2 (9.1)	
Cadre, n (%)			
*Medical doctor*	8 (36.4)	7 (31.8)	0.600
*Midwife*	8 (36.4)	6 (27.3)	
*Nurse*	5 (22.7)	9 (40.9)	
*Others*	1 (4.6)	0 (0.0)	
No. of births assisted per month (times), x̄ (SD)	7.8 (4.4)	4.4 (3.0)	0.002
*≤5*	9 (40.9)	16 (72.7)	0.024
*>5*	13 (59.1)	6 (27.3)	
Experience of managing a non-breathing baby, n (%)			
*No*	1 (4.6)	4 (18.2)	0.238
*Yes*	21 (95.5)	18 (81.8)	
			
Level of confidence in supporting childbirth (0–5 scales), x̄ (SD)	3.5 (0.8)	3.2 (1.1)	0.210
*1–3*	7 (31.8)	12 (54.6)	0.118
*4–5*	15 (68.2)	10 (45.4)	
			
Years of experience in maternity care, x̄ (SD)	9.0 (5.5)	13.3 (10.4)	0.081
*<10*	17 (77.3)	10 (45.5)	0.028
*≥10*	5 (22.7)	12 (54.5)	

n – number of participants, SD – standard deviation, x̄ – mean

**Table 2:** Knowledge and skill baseline scores

Variables	Total score	Intervention		Control		*P*-value (Wald test)
		**n**	**x̄ (SD)**	**n**	**x̄ (SD)**	
Knowledge score	18	22	14.8 (2.3)	22	16.4 (1.4)	0.153
Skill score (total)	106	21	100.0 (4.5)	22	100.6 (4.2)	0.770
Skill score (breathing baby)	44	21	42.5 (1.3)	22	42.8 (1.3)	0.565
Skill score (non-breathing baby)	62	21	57.5 (3.6)	22	57.8 (3.5)	0.834

n – number of participants, SD – standard deviation, x̄ – mean

The response rate for the weekly implementation status survey was 33.3% (84 responses over 252 weekly surveys in three months). In the control arm, one district hospital reported conducting a monthly practise session on management of a breathing baby only, without facilitator feedback.

### Primary outcomes

The mean knowledge and total skill scores at endline were 14.4 (SD = 2.5) and 91.5 (SD = 9.2) in the intervention arm and 15.5 (SD = 2.2) and 88.1 (SD = 9.5) in the control arm (**Table 3**). The endline knowledge and total skill scores were 0.59 (95% confidence interval (CI) = −1.40, 2.52) points and 3.67 (95% CI = −2.90, 10.24) points higher in the intervention arm than the control arm, after adjusting for corresponding baseline score.

**Table 3:** Endline test score difference between the two arms using a linear mixed effects model*

Test	Intervention		Control		Mixed model (no imputation) (n = 37)		Alternative mixed model (no imputation) (n = 44)†		Single imputation: BVCF (n = 44)†		Multiple imputation: PMM (n = 44)†	
	**n**	**x̄ (SD)**	**n**	**x̄ (SD)**	**Coefficient**	**95% CI**	**Coefficient**	**95% CI**	**Coefficient**	**95% CI**	**Coefficient**	**95% CI**
Knowledge score	20	14.4 (2.5)	17	15.5 (2.2)	0.59	−1.40, 2.52	0.45	−1.29, 2.19	0.37	−1.29, 2.03	0.46	−1.37, 2.29
Skill score (total)	20	91.5 (9.2)	17	88.1 (9.5)	3.67	−2.90, 10.24	3.95	−1.42, 9.31	1.61	−3.28, 6.51	3.29	−2.69, 9.27
Skill score (breathing baby)	20	39.7 (4.4)	17	39.2 (4.3)	0.80	−1.83, 3.44	0.75	−1.82, 3.32	0.11	−2.24, 2.48	0.50	−2.11, 3.11
Skill score (non-breathing baby)	20	51.9 (6.1)	17	48.9 (6.9)	2.89	−2.02, 7.81	3.20	−0.69, 7.10	1.35	−2.24, 4.93	3.12	−1.37, 7.60

BVCF – baseline value carried forward, CI – confidence interval, PMM – predictive mean matching, x̄ – mean

*All models are adjusted for the corresponding baseline scores.

**†**One provider was removed from analysis of skill scores due to missing data in baseline values.

The endline skill scores for management of a breathing baby and non-breathing baby were 39.7 (SD = 4.4) and 51.9 (SD = 6.1) in the intervention arm and 39.2 (SD = 4.3) and 48.9 (SD = 6.9) in the control arm (**Table 3**). The endline skill scores for management of a breathing baby and non-breathing baby were 0.80 (95% CI = −1.83, 3.44) points and 2.89 (95% CI = −2.02, 7.81) points higher in the intervention arm than the control arm, after adjusting for a corresponding baseline score. Focus group discussions also found that both facilitators and providers believed that continuous practise would have improved their clinical skills and team performance. Many providers reported having high level of confidence in providing newborn care, including management of non-breathing baby (Table S1 in the **Online Supplementary Document**).

The results were consistent across different models, including mixed model, alternative mixed model and multiple imputation. Estimations became small for all outcomes in the single imputation with baseline value carried forward. Similar results were obtained after adjusting for years of experience in maternity care and number of births assisted per month (Table S2 in the **Online Supplementary Document**).

### Acceptability and feasibility to the intervention

Both district health care providers and facilitators had positive attitudes towards the intervention (Table S1 in the **Online Supplementary Document**). Most felt that provider’s skills needed to improve in order to optimise newborn health outcomes, and the intervention was well-designed to achieve this. District-level providers valued the involvement of central and provincial facilitators, particularly in getting more regular feedback. Facilitators felt proud to contribute to an intervention that would improve newborn health outcomes. They particularly appreciated the resource-saving aspect of remote supportive supervision, compared to on-site supervisory visits. They felt it was feasible to send feedback by WhatsApp instead of travelling to each district hospital, which was expensive and time-consuming. Notwithstanding the positive views of the intervention, some participants also experienced stress in continuing the intervention: providers felt they had to be ready to perform well, manage time for practise and sometime regret not being able to practise as planned.

Despite of the positive view of the intervention, 21 providers in the intervention arm submitted videos of a total 63 sessions practising management of breathing babies, and 61 sessions practising management of non-breathing babies in three months (about two sessions per month), which was fewer than expected. The implementation status survey showed that the median time spent practicing in the three-month period was 30 minutes (Interquartile range = 89), ranging from 7–500 minutes. In FGDs, providers reported that the main burden of using the intervention was the amount of time they had to spend practising and recording videos (Table S1 in the **Online Supplementary Document**). A group practise session approach was also difficult, as there was limited time for multiple providers to meet, meaning they had to find practise time outside of working hours. Time constraints were particularly challenging for providers who did not receive a salary (*i.e*. working voluntarily in public hospital services). They preferred to use their spare time to earn money. They felt it would be difficult to continue without financial incentives to compensate them.

According to the data stored in WhatsApp, all videos shared in the group received feedback comments from provincial or central facilitators. Focus group discussions showed that facilitators appreciated that the intervention could be done around their other commitments. Facilitators found the videos helpful and believed that their feedback would help improve providers’ skills. Unlike on-site supervisory visits, they could review videos and provide feedback as they became available. Facilitators largely viewed this flexibility positively. However, this could lead to delayed responses in the WhatsApp group, meaning the communication between facilitators and providers was perceived be somewhat inconsistent.

Both facilitators and providers showed willingness to continue the intervention, however, they were not confident enough to do so due to time constraint (Table S1 in the **Online Supplementary Document**). Providers also justified skipping some important EENC steps such as hand washing while practising. Some facilitators stated that self-practising the management of a non-breathing baby needed to be more realistic, in order to effective in real-world situations.

### Facilitators and barriers to implementing the intervention

Providers stated that all equipment necessary for practise was available (Table S1 in the **Online Supplementary Document**). Commitments and support from hospital leadership encouraged providers to find time and continue practise. For example, the hospital director offering staff a practise room and dedicated time to practise was found to be helpful. Providers indicated that feedback from facilitators kept them learning, whereas facilitators were mainly motivated to help save children’s lives. A few facilitators and providers felt that financial support would be an enabler, particularly for internet data – they sometimes paid for their own data to send and receive videos when the internet at hospital was not functional.

Barriers included a relatively small number of experienced facilitators at provincial hospitals, which made it difficult to provide timely feedback (Table S1 in the **Online Supplementary Document**). Facilitators also felt uncomfortable sending feedback by message due to limited skills and experience in online supervision or using mobile phone while on duty, which contributed to delays in sending feedback. Poor internet access was a consistent barrier – each hospital had an internet connection, but it was often unreliable, meaning they had to purchase their own mobile data. The intervention encouraged providers to use their own phone, however some of their phones did not have enough memory to record videos.

## DISCUSSION

We designed and pilot-tested a novel intervention that combines repeated self-practise and mobile phone-based supportive supervision to improve neonatal care provider’s knowledge and skills in resource-limited settings. We found higher skill scores in the intervention arm, suggesting the potential for effectiveness of this intervention to maintain health care providers’ skills. Both providers and facilitators responded positively about the intervention, with reports of perceived improvement in providers' clinical skills. However, time constraints were reported as the main barrier to continuing this intervention.

The result showed that a mean difference in the skills score on management of a non-breathing baby between two arms was 2.89 units (6.2% improvement in score). The number of births attended each month was higher in the intervention than in the control arm, which could potentially improve skills in the intervention arm at endline. However, there was only one case of a non-breathing baby reported during the study period, so the number of births would not have influenced the skills in managing a non-breathing baby, which showed the greatest difference at endline. Providers in the intervention arm had fewer years of experience than those in the control arm, which may also have influenced the better skills in the intervention, as younger providers are known to implement skills more consistently than older providers [25]. However, the adjusted analysis showed the consistency of the results with the unadjusted analysis. Improvements were mainly observed in bag and mask handling, indicating the potential impact of the intervention on neonatal health outcomes and the need for further evaluation to confirm effectiveness.

Low birth volume at the health facility has been reported to be associated with low quality of care [26]. All district hospitals included in our pilot trial had the low birth volume with mean number of childbirths assisted by each provider ranging from 4.1–8.7 per month. It is noteworthy that the difference in the simulation test score at endline between the arms was greater for management of a non-breathing baby than for management of a breathing baby. This may be because health care providers had more opportunities to assist with breathing babies than non-breathing babies while they were on duty. The results support our hypotheses that repeated practise may be particularly useful for improving the management of non-breathing babies, which are relatively rare to encounter in practice. Regardless of the retention of resuscitation skills, we observed a low proportion of providers achieving chest rises within one minute of birth in both arms: only two providers in the intervention arm and none in the control arm were able to achieve this at endline. The chest rise achievement within one minute of birth was low even at baseline. During the FGDs, it was mentioned that the self-practise was not performed in a realistic manner; district health care providers tended to do it slowly so as not to miss any steps. To prevent mortality and morbidity from asphyxia, it is essential to achieve an effective chest rise within one minute of birth [27]. A study in Tanzania showed that each 30-second delay in initiating face mask ventilation increased the risk of deaths and adverse outcomes by 16% [28]. Future EENC coaching and self-practise should focus not only on steps but also on timeliness of care to ensure that chest rise is achieved within one minute. Providers should also be encouraged to time their performance during a self-practise.

In our pilot trial, most providers attended two sessions per month, once each for the management of a breathing and non-breathing baby, while it was initially expected to be two sessions in two weeks. Notwithstanding providers’ positive attitudes towards the intervention, time constraints inhibited more frequent practise. Our FGDs suggested that more frequent practise may not be feasible and may reduce providers' motivation. It has been reported that low-dose, high-frequency training sessions conducted five and more in nine months was associated with better clinical competence [29]. Our intervention also showed the potential effectiveness with the monthly practise (practise two sessions per month) and was considered to be more feasible. Therefore, a monthly practise model can be considered for further evaluation. To overcome the identified barriers to group practise, it would be useful to set up a practise corner so that practise can only be carried out by one provider. We also found that facilitator feedback and commitment of hospital leadership helped maintain motivation of providers to find time for practise. This is consistent with a systematic review in LMICs reporting that a supervision component has a positive effect on provider motivation [30]. To enable timely feedback from facilitators, additional facilitator training to increase the number of facilitators and confidence in online supervision would be required. In our pilot trial, one district hospital managed to maintain participation of all providers in practice as district hospital director allocated time to practise. In a future study, it would be critical to formally involve the hospital leadership in the intervention and discuss how to integrate self-practise into the daily routine. Monetary incentives could maintain participation, but on the other hand, the effectiveness of provider incentives on quality care improvement was reportedly uncertain [31]. Incentives have a sustainability issue and need to be implemented carefully, while the intervention also needs to find a way to compensate the cost of mobile data.

Notwithstanding the barriers, this intervention has the potential to maintain clinical skills and is well accepted and considered more feasible than frequent supervisory visits, which are more costly and time-consuming to implement. This warrants a large trial to evaluate the effectiveness of the intervention. A future trial should incorporate a strategy to engage facility leadership to arrange time to participate, improve provider motivation and ensure the sustainability. Cost-effectiveness will also provide useful information for decision-makers to consider this intervention. If proven effective, this intervention may be applicable in other countries with limited resources where there is a good mobile network coverage, as it could be implemented without additional investment.

### Strengths and limitations

This study was conducted in collaboration with the Ministry of Health in Lao PDR, which allows us to design the intervention to fit in the context. This engagement strengthens feasibility and sustainability of the intervention. The findings can be used to improve the intervention before it is tested in a larger trial. The study methods and findings are generalisable to a definitive trial, as it targets district hospitals in the similar settings. The follow-up rate was high at 84% (37 out of 44 participants) and the bias due to loss to follow-up would be minimal. The limitation is the small size of the trial. It is underpowered to test the effectiveness of the intervention. However, our primary aim was to estimate a potential effective size for a future definitive trial and to assess the feasibility and acceptability of the intervention, therefore, this pilot study has served its purpose. The small sample size also made it difficult to ensure comparability between the two arms, despite our selection of similar district hospitals within the same province. Although the results were compatible after adjusting for baseline variables, there may be other unmeasured factors that influenced the outcome. A larger trial will be able to control for these unmeasured factors. Also, the implementation status, such as time and frequency of self-practise, relied on self-reporting, and it may have been under or over reported. Another limitation is contamination as masking of providers was not possible due to the nature of the intervention. However, the intention to treat analysis showed a potential improvement in skills in the intervention arm compared to the control arm. This supports the promising effect of the intervention in the real-world situation.

## CONCLUSIONS

This pilot cluster-randomised trial evaluated a novel intervention combining self-practise and mobile phone-based support supervision to improve the knowledge and skills of newborn care providers in a resource-limited setting. The intervention appears promising over a three-month period. The intervention was generally well accepted by providers and facilitators, who perceived an improvement in provider clinical skills. Major barriers included time constraint and facilitators’ limited experience in supervision via mobile phones. A larger trial to assess the intervention’s effectiveness is warranted with measures to address the barriers such as the creation of practise corners, leadership involvement and facilitator training. Future research should consider its effects on neonatal health outcomes, as well as its cost-effectiveness in order to assess scalability and sustainability.

## Additional material:


Online Supplementary Document
