# Supplementary material for: An innovative, digital approach to training district health care providers on essential newborn care skills: findings from a pilot cluster-randomised trial in Lao People’s Democratic Republic
Source: J Glob Health. 2025 Jun 2;15:04163. doi: 10.7189/jogh.15.04163 (PMC12127832; doi:10.7189/jogh.15.04163)
Supplement: Online Supplementary Document [file jogh-15-04163-s001.pdf]

Supplement to: Horiuchi S, Smith R, Phrasithideth B, Kounnavongsa O, Thilakoune C, Xayavong H, Sakamaki K, Rattana S, Vogel JP. An innovative, digital approach to training district healthcare providers on essential newborn care skills: findings from a pilot cluster-randomised trial in Lao People's Democratic Republic. J Glob Health. 2015;15:04163.

Table S1. Themes and subthemes: Acceptability, facilitators and barriers to implementing the intervention

Theme	Subthemes	Quotes
A. A valuable and feasible intervention.	1. Improving technical skills and newborn health outcomes were viewed positively by staff.	"I feel happy. In the past, we have only studied the theory, and we have not experienced the real cases sometimes. We have only practiced with case scenarios." (district healthcare provider)<br><br>"First of all, I feel very happy to have participated in the remote supportive supervision on EENC for the district hospital staff. Even though we didn't actually visit them, we saw them through online media." (provincial facilitator)<br><br>"Learning is not just to learn one time. It's a continuous process and doing practice. It's good, non-breathing case is rare, but it's important because this can lead to the death of the child. If they (healthcare providers) could not do the assessment, they could not help babies…" (central facilitator)
	2. District-level providers appreciated the regular communication with facilitators, and feedback in the chat group was generally acceptable.	"I would like to share that it is the first time for me to do video recording, it is exciting and it's like acting. It is good that we could practice, and they (facilitators) commented us when there's something correct or incorrect. It's like we trained ourselves." (district healthcare provider)
	3. Facilitators appreciated the intervention as it did not require a budget for implementation, such as onsite supervisory visits.	"Another benefit is that we can save our budget from going down to the district levels with cost of car fuel and per diem for trainers. In contrast, this mobile-based supportive supervision works well, they (district healthcare providers) just film a video and send it to provincial hospital staff for comments." (provincial facilitator)<br><br>"In the past, the ministry did it (on-site supervision) in 3 months, 6 months, but there's no money, isn't there... The outreach supervision needs a lot of budgets, like transportation from the central to the provincial level." (central facilitator)
	4. Both district-level providers and facilitators felt pressure to continue with the intervention as they were constantly seeing messages posted on the group chat.	"I feel pressured. It's as if we have someone watching us every time, we think about how to pass or how to perform. We have to be ready, which we have to try and practise. Make more videos and ask for comments." (district healthcare provider)<br><br>" Both happy and unhappy feelings". "We have pressure on us to do it, they already said that we have to upload one video a week, but we don't

		have time to do it. We regret why we didn't add a video.” (district healthcare provider)<br><br>“It’s a bit harsh, but we have made our mind to make it happen.” (provincial facilitator)
B. Struggling to find time to practise.	1. District providers struggled to find time to practise and spent a lot of personal time participating in the intervention.	“I think it (the intervention) interfered with some works, like the work for mother and child health, it does cover not only vaccination in 24 villages, but also nutrition, child development and pregnancy screening, which must be done daily. We also have shifts. By the end of the month, I have to make a summary report and be responsible for all the work related to vaccination.” (district healthcare provider)<br><br>“We spent our free time recording videos. Sometimes we wanted to do something else, like personal things. But we had to use our free time to make this video.” (district healthcare provider)
	2. Use of personal time for participating in the intervention conflicted with family commitments.	“I would like to tell you the truth about my feelings. It's about the fact that we come to work as we are not yet official staff (volunteers). When we have time, we come to practise, but we don't have that time. We want to use that time to deal with family matters, money matters, like selling lottery tickets.” (district healthcare provider)
	3. Facilitators appreciated the time flexibility and time saving feature of the intervention, which facilitated their participation. But this flexibility also led to the delayed response to district-level providers.	“When they (district healthcare providers) film a video clip and send it, we don’t need to watch it at that moment to make comments. We use our spare time to watch the video. I think it’s good to do this. We don’t waste much time. For example, during the day, we are working for patients, when we are at home or when have spare time, we can watch and comment or say hello; it’s good, I’m happy.” (provincial facilitator)<br><br>“Sometimes they took several days to reply, the provincial and central teams may have lots of work like we do. If we have sent it to them, they will reply when they’re available.” (district healthcare provider)
	4. Limited time hindered regular practice and timely feedback, limiting effective interaction between district-level providers and facilitators.	“If they (district healthcare providers) are free, they sent almost every day. If they are not free, only 2 or 4 videos because they work as a team or pair.” (provincial facilitator)<br><br>“Now, when we see the videos that have been sent, we wait for the province to see them and comment on them...but they didn't comment...so

		sometimes we had to ask if we could comment before the Province...it's like this.... For the videos, it depends on when the district sends them... assuming they send them today .... the other day...... if there is a schedule, it would be good; we could set our own schedule." (central facilitator)
C. Participants' beliefs in the intervention and confidence in behaviour change.	1. Facilitators and district-level providers believed that continuous practice would improve their clinical skills, but had mixed opinions about the effectiveness of the mobile-based supervision.	"The staff might do wrong things, for example, they cannot put the bag & mask firmly, so the wind does not go in properly; to adjust the position of the mannequin and the real patient is different...... we need to hold the chin in the right position and 'mask seal' to blow the wind in... but for the case of real patient, it is not like in theory. It is very difficult (in real patient than doll). We need to practise more." (district healthcare provider)<br><br>"Better to have someone come. For example, to come at least once or twice a year to supervise....We want them (the facilitators) to be open with us. For example, what we are improving, what we are still doing wrong, we remember that." (district healthcare provider)<br><br>"In order to improve the quality of this work and to do it on an ongoing basis, we may need more supervision for them and for us. Personally, I think it would be great to do outreach supervision, to supervise in the field while they are practising. It could be quarterly supervision. Let's say once in three months or six months. Mobile supervision is not the same as what we can see during practice on site, which would be more focused." (provincial facilitator)
	2. District providers and facilitators felt the intervention had positive influences on district providers' knowledge and skills, teamwork, and sense of confidence in providing care	"It (the intervention) helps to be better, and I feel more confident when there's a case of non-breathing or foetal distress. If it happens, there is a better chance that the child will survive and be safe.…. Everyone is confident and we have confidence in the team." (district healthcare provider)<br><br>"When the baby comes out, in the case of a non-breathing baby, it is difficult to help both the baby and the mother at the same time, it would be too hard; if we have colleagues to join to work together, unity, we will not feel tired." (district healthcare provider)

		“They (district health workers) will gain a lot of skills because they practise the work every day. ...for the 1st and 2nd video, I felt that they were not doing very well. After we gave them feedback, we saw that they improved a lot.” (provincial facilitator)<br><br>“When we watch videos from time to time, we see that they (district healthcare providers) are improving and getting better, they can do better. I think through our supervision we can say that we are confident that they will probably be able to handle it when they have cases.” (central facilitator)
	3. The use of simulation in the intervention was seen to be different from real-world practice, and that there was still a “know-do” gap.	“The goal we have at work is that the child must be safe, and the steps may not go as we practise with the manikin. There’ll not be order for each step, but everything is done correctly to keep the child safe. But we did not have time to follow the steps such as washing hands, like 7 steps, wearing gloves, please wait for a second (say a joke). It isn’t like that; we have to hurry.” (district healthcare provider)<br><br>“I want them (district healthcare providers) to focus on the non-breathing case. During the assessment visit they were in a panic. They don't know how important it is to resuscitate within 1 minute. Delayed resuscitation means difficulty in helping the child. ....Based on our assessment of non-breathing cases, they are too slow to resuscitate. We want them to be more skilled and we want the provincial facilitators to focus on this. If they don't have patients, what can they do... so they don't forget the lesson?” (central facilitator)
	4. Facilitators and district providers were willing to continue the intervention, but district providers felt they lacked time to do so.	“I want to practise my skills more often, to understand myself in the points that are often forgotten. So that when we see patients we don't forget so easily and don't panic too much. Practise to be better. If we can't do something, we'll be able to do it if we practise every day, those are the good points.” (district healthcare providers)<br><br>“We don't want to do filming because we don't have time.” (district healthcare providers)<br><br>“I want to continue. But as the doctor said it would be difficult for us to do so due to limited time.” (district healthcare provider)

		"I think we should continue with this activity. I think if the district staff are motivated to continue, they will develop and improve their skills. When they have better skills, they will be more motivated. .... If this activity continues, they will improve their skills. Of course, if they have better skills, more people will come to use the service... So those who facilitate, the provincial health department, the director of the hospital, to encourage, to motivate them to continue working on this." (provincial facilitator)
D. Facilitators for implementing the intervention.	1. Environmental factors - ensuring equipment was available, and commitment of hospital leadership to the intervention – encouraged self-practise amongst providers.	"The mannequin is with us. If we have free time, we can come and practise with it." (district healthcare provider)<br><br>"It (self-practise) did not interfere with other work. Sometimes the deputy director asked us to do it (self-practise) .... Do it once, do it twice .... she is the deputy head of the maternity unit; she works actively and enthusiastically; this work was considered important." (district healthcare provider)
	2. Receiving facilitators' comments helped district providers to keep learning.	"I felt happy and proud because the provincial facilitators paid attention to us and let us practise solving the problem in our hospital. We don't know what will happen to the hospital next time, so everyone must try to practise." (district healthcare provider)
	3. Desire to improve and serve people drove the participation of facilitators.	"The reason why we have to watch the videos is because we want to support the district hospital staff to improve their skills and use them in their work to reduce maternal and child deaths, increase the safety of the child and help the child when needed. If they don't have the skills or are using the wrong skills, we need to teach them so they can develop. Even though we have to invest time, our participants can save children." (provincial facilitator)
	4. Both providers and facilitators requested financial incentives for better participation and continuation of the intervention.	"As I said, it would be good if there was a motivational incentive. For the skills, if we do not practise, we may forget them." (district healthcare provider)<br><br>"providing small gift… if possible… like top up telephone card… we do not request much… for motivation… or are there anything else… better than this will be OK…" (provincial facilitator)

E. Barriers for implementing the intervention.	1. Facilitators were sensitive to district-level providers' feelings, and did not feel confident about giving feedback via chat message.	"For example, when we see them not doing it right and we want to help them improve. But typing does not work as well as real practice... well, the baby is out, you have to press like this, hold like this, press... this is an obstacle when we have to explain to them." (central facilitator)<br><br>"it's like typing and deleting messages many times and see if it is appropriate to say." (central facilitator)
	2. There was limited number of trained facilitators, which sometimes delayed a response to district-level providers.	"There are very few senior facilitators as we said. We need to train new ones. …"
	3. Facilitators found it difficult to use the mobile phone in the workplace, therefore had to provide feedback during their free time.	"For example, if I receive a video and watch it during working hours. Others such as our colleagues or patients might think that I am not working, just playing on the phone…" (provincial facilitator)
	4. Poor internet access at workplaces and device issues limited timely sharing and viewing of videos.	"Sometimes, we can't use the (phone) camera, there is little space on the memory card, then the app does not respond." (district healthcare provider)<br><br>"Sometimes I borrow the Internet package and then I have to do the payback." (district healthcare provider)<br><br>"Internet is the problem… we need to top up by ourselves…" "We, central or provincial facilitators, really need to use the internet... sometimes when we watch the video it stops halfway through because we have no credits." (provincial facilitator)

Table S2. Endline test score difference between the two arms adjusted for baseline covariates

Test	Mixed model (no imputation) (n = 37)		Alternative mixed model (no imputation) (n = 44) [1]		Single imputation: BVCF (n = 44) [1]		Multiple imputation: PMM (n = 44) [1]	
	**Coefficient**	**95% CI**	**Coefficient**	**95% CI**	**Coefficient**	**95% CI**	**Coefficient**	**95% CI**
Knowledge score	0.78	-1.63–3.19	0.59	-1.12–2.30	0.88	-1.10–2.85	0.71	-1.49–2.90
Skill score (total)	5.34	-0.11–10.81	4.12	-1.22–9.46	3.75	-0.37–7.88	5.04	-1.14–11.22
Skill score (breathing baby)	2.14	-0.60–4.89	0.66	-1.89–3.22	1.66	-0.28–3.60	2.09	-0.69–4.86
Skill score (non-breathing baby)	2.61	-2.43–7.65	3.44	-0.36–7.24	1.60	-1.82–5.02	2.81	-1.51–7.13

BVCF: baseline value carried forward; PMM: predictive mean matching; CI: confidence interval
All models are adjusted for the corresponding baseline scores, years of experience in maternity care and number of births assisted per month.
[1]One provider was removed from analysis of skill scored due to missing data in baseline values.
